# Persistently Transmitted Viruses Restrict the Transmission of Other Viruses by Affecting Their Vectors

**DOI:** 10.3389/fphys.2018.01261

**Published:** 2018-10-01

**Authors:** Gong Chen, Qi Su, Xiaobin Shi, Huipeng Pan, Xiaoguo Jiao, Youjun Zhang

**Affiliations:** ^1^College of Plant Protection, Hunan Agricultural University, Changsha, China; ^2^Department of Plant Protection, Institute of Vegetables and Flowers, Chinese Academy of Agricultural Sciences, Beijing, China; ^3^Hunan Provincial Key Laboratory for Biology and Control of Plant Diseases and Insect Pests, Hunan Agricultural University, Changsha, China; ^4^College of Agriculture, Yangtze University, Jingzhou, China; ^5^Hubei Collaborative Innovation Center for Green Transformation of Bio-Resources, College of Life Sciences, Hubei University, Wuhan, China

**Keywords:** persistently transmitted virus, vector transmission, ecological interaction, adaptive manipulation, pathogen competition

## Abstract

Diverse pathogens, plant hosts, insect vectors, and non-vector herbivores coexist and interact in natural systems. An example is the cooccurrence of insects *Bemisia tabaci* Q and *Frankliniella occidentalis* and the pathogens tomato yellow leaf curl virus (TYLCV) and tomato spotted wilt virus (TSWV) on the same plant. In addition, both TYLCV and TSWV are persistently transmitted in these insect species. However, TSWV reduces the fitness of *B. tabaci* Q; therefore, we investigated whether TSWV affects the transmission of TYLCV to tomato. Both TYLCV and TSWV are persistently transmitted. Although *B. tabaci* Q cannot transmit TSWV, we found that this insect species is able to acquire and retain this virus serotype, indicating that the effects of TSWV on TYLCV transmission in the current study result from effects on the vector. The acquisition, retention, and transmission of TYLCV by *B. tabaci* Q were reduced when the insect vector contained TSWV. Additionally, the TYLCV acquisition and transmission by *B. tabaci* Q were reduced when the host plant was inoculated with TSWV before TYLCV or simultaneously with TYLCV. We also found that *F. occidentalis* fecundity and transmission of TSWV were reduced when *F. occidentalis* contained TYLCV. Our findings are consistent with the hypothesis that persistently transmitted viruses can restrict the transmission of other viruses by affecting their insect vectors.

## Introduction

Research has increasingly indicated that vector-borne parasites can manipulate some phenotypes of their vectors or hosts in ways that enhance parasite transmission (Lefèvre and Thomas, 2008; Mauck et al., 2012; Blanc and Michalakis, 2016). Pathogen-induced changes in host-plant phenotypic traits often result in increased attraction of vectors to infected host plants or in other changes in vector performance on infected host plants; the effects of these changes in the host on the interactions between the vector and pathogen can be mutualistic, neutral, or antagonistic (Hammond and Hardy, 1988; Fereres and Moreno, 2009). Pathogen-induced changes in vectors can strongly affect transmission efficiency and vector fitness and can thus have significant effects on agricultural and other ecosystems and on human health (Ajayi and Dewar, 1983; Hammond and Hardy, 1988; Mauck et al., 2010, 2012). The interactions between persistently transmitted viruses and vectors have often been studied, but research is lacking on the interactions between persistently transmitted viruses and non-insect-vectors (Su et al., 2016). The persistently transmitted viruses require vectors to feed on an infected host for a sustained period to acquire and circulate (and sometimes replicate) virions, and then disperse to a new, healthy host (Mauck et al., 2012).

In natural systems, the evolution of pathogen effects on host characteristics, including those that influence the interactions with insect vectors, is likely to be influenced by complex ecological interactions involving host plants, insect vectors, non-vector herbivores, and multiple pathogens (Salvaudon et al., 2013). For example, pathogen-induced changes in a host-plant phenotypic trait that enhance insect-vector recruitment may also increase pathogen transmission via increased herbivory. In addition, parasite-induced changes in herbivores that directly or indirectly affect herbivore fitness may influence parasite transmission (Pan et al., 2012). The existing literature shows that insect vectors prefer to settle on, or at least do not avoid settling on, host plants infected with persistently transmitted viruses and that insect vectors show no preference or reduced preference for host plants infected with non-persistently transmitted viruses relative to healthy plants (Ingwell et al., 2012; Mauck et al., 2016, 2012). Moreover, persistently transmitted viruses typically enhance host plant quality for insect vectors, resulting in increased insect vector longevity, fecundity, or survival. In contrast, non-persistently transmitted viruses usually reduce or have no influence on host plant quality (Mauck et al., 2012).

Although increases in insect vector fitness caused by persistently transmitted viruses have been extensively studied (Mauck et al., 2012), the influence of persistently transmitted viruses on non-vector insects have seldom been investigated. Just as host plants may be simultaneously fed upon by non-vector and vector herbivorous insects, they may be simultaneously infected by multiple viruses, and such multiple infections of a single host plant are likely to occur in the field. As a consequence, herbivorous insects may simultaneously ingest several pathogens, and we have observed simultaneous outbreaks of tomato yellow leaf curl virus (TYLCV), tomato spotted wilt virus (TSWV), *Frankliniella occidentalis*, and *Bemisia tabaci* in the same greenhouse. Previous studies have documented a mutualistic relationship between TYLCV and *B. tabaci* Q (Pan et al., 2012; Chen et al., 2013). In addition, our previous results showed that TSWV can reduce the fitness of *B. tabaci* on pepper (*Capsicum annuum* L) (Pan et al., 2013). Many other studies have indicated that parasites can influence the transmission of pathogens by changing the host quality for vectors (Ebbert and Nault, 2001; Taylor and Hurd, 2001; Pan et al., 2012; Liu et al., 2013) or by changing the cues (attractants) used by the foraging insect vectors to locate host plants (Jennersten, 1988; Eigenbrode et al., 2002; Jiménez-Martínez et al., 2004a,b).

Tomato yellow leaf curl virus is a destructive begomovirus of tomato plants and is transmitted by *B. tabaci* in a circulative and persistent manner (Cohen and Nitzany, 1966; Mehta et al., 1994; Caciagli et al., 1995). TSWV is another persistently transmitted virus. Like most tospoviruses, TSWV replicates in its insect vectors (*F. occidentalis* and other thrips) and is transmitted in the vector’s saliva during persistent feeding (Elliott, 1996; Ullman et al., 2002). TSWV also can enhance the fitness of *F. occidentalis* (Maris et al., 2004). Unlike TYLCV, TSWV is not transmitted by *B. tabaci* (Zhao et al., 2015). Similarly, TYLCV is not transmitted by *F. occidentalis*. When these insects feed on host plants that are infected by both TSWV and TYLCV, they are likely to ingest both viruses.

The sweet potato whitefly, *B. tabaci* (Gennadius) (Hemiptera: Aleyrodidae), is a vector of begomoviruses. It causes serious damage in many crops by direct feeding and by vectoring plant viruses. The most current molecular evidence reveals that *B. tabaci* actually includes at least 24 genetically distinct but morphologically indistinguishable cryptic species (De Barro et al., 2011). The most damaging and widespread cryptic species are the Mediterranean genetic group (biotype Q) and the Middle East–Asia Minor 1 genetic group (biotype B). *F. occidentalis* is also an important agronomic pest and has a worldwide distribution (Ullman et al., 1997). It reduces plant growth, deforms plant organs, and causes cosmetic damage in the form of silver scars on leaves and flowers (Kirk and Terry, 2003). The small size and slender shape, short reproductive cycle, high fecundity, and polyphagous feeding behavior of *B. tabaci* and *F. occidentalis* help make them successful invasive species (Brown et al., 1995; Morse and Hoddle, 2006).

As noted earlier, the performance of *B. tabaci* is reduced when it feeds on TSWV-infected pepper plants (Pan et al., 2013). Based on these results, we hypothesized that persistently transmitted viruses can restrict the transmission of other viruses by affecting their vectors. The current study describes how the acquisition of TSWV by *B. tabaci* Q affects the whitefly’s ability to acquire, retain, and transmit TYLCV; how the order in which a host plant is infected with the two viruses affects the whitefly’s ability to acquire TYLCV from that plant; and how the infection of tomato by TSWV affects the whitefly’s ability to transmit TYLCV. We also describe how the acquisition of TYLCV by *F. occidentalis* affects its fecundity and its ability to transmit TSWV.

## Results

### Detection of TSWV RNA in *B. tabaci* Q as Affected by Acquisition Access Period (AAP)

Tomato spotted wilt virus RNA was detected in 40% of the *B. tabaci* Q adults after a 3-h AAP on TSWV-infected pepper leaves (**Table 1**). The percentage of Q adults with detectable TSWV RNA increased as the AAP increased and was 100% with a 24-h AAP.

**Table 1:** Efficiency of TSWV acquisition and retention by *Bemisia tabaci* Q. *B. tabaci* Q adult females were assayed for TSWV RNA after they had a 0- to 48-h acquisition access period (AAP) on TSWV-infected pepper plants.

TSWV acquisition from pepper		TSWV retention on cotton	
	
AAP (h)^a^	Adults with TSWV RNA (%)	Duration of feeding (d)^*a*^	Adults with TSWV RNA (%)
0	0	0	100
3	40	5	100
6	50	10	90
12	90	15	100
24	100	20	50
48	100		


*^*a*^Ten whiteflies were assayed at each time point. After a 72-h AAP, females were transferred to cotton plants, a nonhost for TSWV, and subsequently assayed for TSWV RNA after 0- to 20-d feeding periods.*

### Retention of TSWV RNA by *B. tabaci* Q

All *B. tabaci* Q adult females that probably were infected by TSWV (these adult females had a 72-h AAP) contained detectable TSWV RNA in the 5 d following their transfer onto healthy non-host cotton plants (**Table 1**). Thereafter, the percentage of adults with detectable TSWV RNA ranged from 50 to 100%.

### Acquisition of TYLCV DNA by *B. tabaci* Q Females Carrying TSWV

**FIGURE 1:**
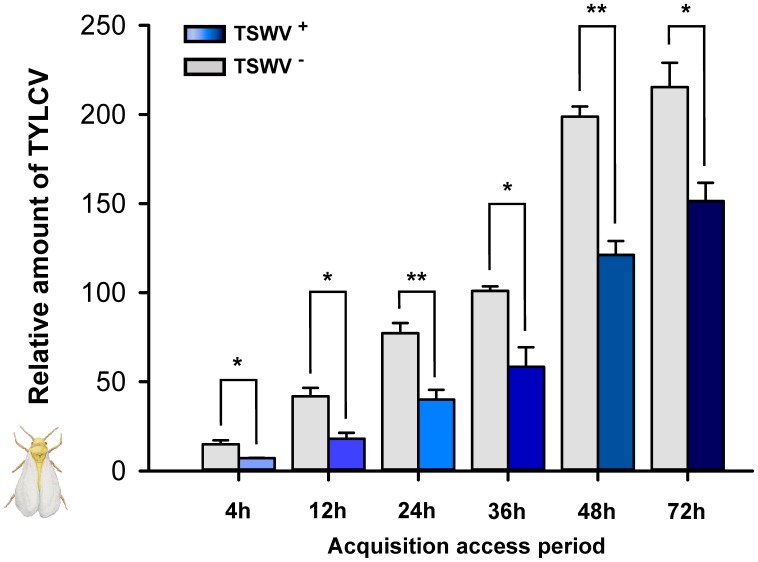
Acquisition of TYLCV DNA by *Bemisia tabaci* Q females as affected by previous exposure of the whiteflies to TSWV. TSWV^+^ or TSWV^-^: *B. tabaci* Q females that had a 72-h acquisition access period (AAP) on TSWV-infected pepper plants or on noninfected pepper plants, respectively, before they fed on TYLCV-infected tomato plants. The *B. tabaci* Q used for TSWV^+^ were assumed to contain TSWV based on **Table 1**. Values are means ± SE. Asterisks indicate significant differences between TSWV^+^ vs. TSWV^-^ treatments (one-way analysis of variance, ^∗^*P* < 0.05, ^∗∗^*P* < 0.01).

Tomato yellow leaf curl virus acquisition was significantly lower for *B. tabaci* Q females that had previously fed on TSWV-infected pepper plants rather than on healthy pepper plants (**Figure 1**). For all treatments, TYLCV DNA reached maximal viral loads in *B. tabaci* Q females after a 48-h AAP.

### Acquisition of TYLCV DNA by *B. tabaci* Q Females From Tomato Plants Inoculated in Different Order With Both TSWV and TYLCV

**FIGURE 2:**
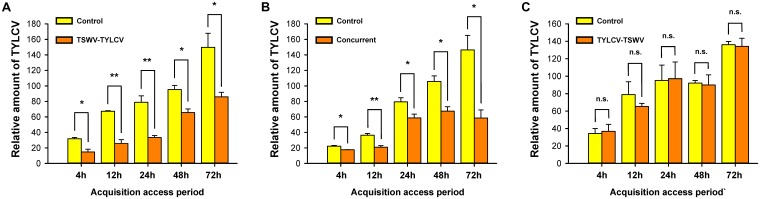
Acquisition of TYLCV DNA by *B. tabaci* Q females from tomato plants inoculated in different order with both TSWV and TYLCV. TSWV-TYLCV: plants were inoculated with TSWV and 2 weeks later with TYLCV. TYLCV-TSWV: plants were inoculated with TYLCV and 2 weeks later with TSWV. Concurrent: plants were simultaneously inoculated with both viruses. Control: plants were inoculated with TYLCV and were mock-inoculated 2 weeks later. **(A)** Acquisition ability of TYLCV DNA by *B. tabaci* Q females from TSWV-TYLCV plants. **(B)** Acquisition ability of TYLCV DNA by *B. tabaci* Q females from concurrent plants. **(C)** Acquisition ability of TYLCV DNA by *B. tabaci* Q females from TYLCV-TSWV plants. Values are means ± SE. Asterisks indicate significant differences between treatments vs. control (one-way analysis of variance; ^∗^*P* < 0.05, ^∗∗^*P* < 0.01, and n.s. indicates not significant, *P* > 0.05).

Relative to TYLCV acquisition by *B. tabaci* Q females on tomato plants inoculated only with TYLCV (Control, **Figure 2A**), TYLCV acquisition was significantly lower on plants that had been first inoculated with TSWV and then with TYLCV (TSWV-TYLCV, **Figure 2B**) or that had been concurrently (Concurrent, **Figure 2C**) inoculated with both viruses (**Figure 2**). TYLCV acquisition, however, did not significantly differ on control tomato plants and tomato plants that had first been inoculated with TYLCV and then with TSWV.

### Retention of TYLCV DNA by *B. tabaci* Q Females Carrying TSWV

**FIGURE 3:**
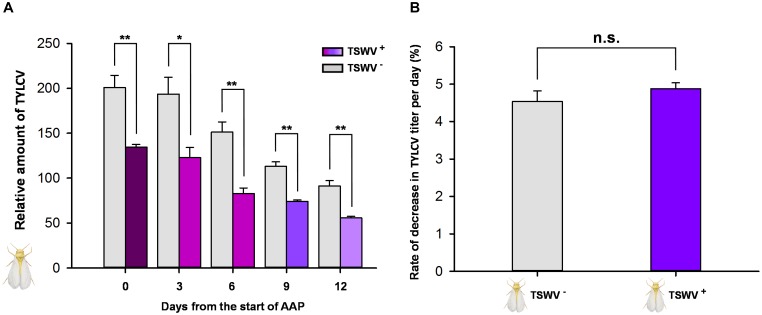
Retention of TYLCV DNA by *B. tabaci* Q females after transfer to nonhost (cotton) plants as affected by previous exposure of the whiteflies to TSWV. TSWV^+^ or TSWV^-^: *B. tabaci* Q females that had a 72-h AAP on TSWV-infected pepper plants or on noninfected pepper plants, respectively, before they fed on TYLCV-infected tomato plants. The *B. tabaci* Q used for TSWV^+^ were assumed to contain TSWV based on **Table 1**. **(A)** Relative quantity of TYLCV retained. **(B)** Rate at which TYLCV titer decreased in *B. tabaci* Q females. Values are means ± SE. Asterisks indicate significant differences between TSWV^+^ vs. TSWV^-^ treatments (one-way analysis of variance; ^∗^*P* < 0.05, ^∗∗^*P* < 0.01, and n.s. indicates not significant, *P* > 0.05).

As indicated by q-PCR, the relative amount of TYLCV on day 0, when the *B. tabaci* Q females were transferred to cotton plants, was lower in females previously exposed to TSWV (they had fed on TSWV-infected pepper plants before they fed on TYLCV-infected tomato plants) than in females not exposed to TSWV (**Figure 3A**). The TYLCV titer decreased gradually in all treatments but was always lower in females that presumably contained TSWV than in females that did not (**Figure 3A**). The rate at which the TYLCV titer declined did not significantly differ between females that were or were not exposed to TSWV (*F*_1,4_ = 1.101, *P* = 0.353) (**Figure 3B**).

### Transmission of TYLCV to Tomato by *B. tabaci* Q Females Carrying TSWV

**FIGURE 4:**
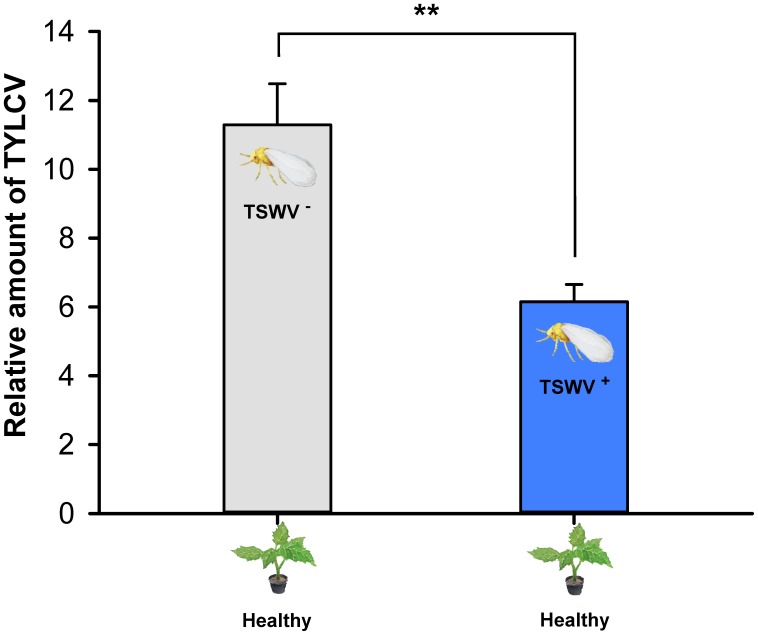
Transmission of TYLCV DNA to tomato plants by *Bemisia tabaci* Q females as affected by previous exposure of the whiteflies to TSWV. TSWV^+^ or TSWV^-^: *B. tabaci* Q females that had a 72-h AAP on TSWV-infected pepper plants or on noninfected pepper plants, respectively, before they fed on tomato plants. The *B. tabaci* Q used for TSWV^+^ were assumed to contain TSWV based on **Table 1**. Values are means ± SE. Asterisks indicate significant differences between TSWV^+^ vs. TSWV^-^ treatments (one-way analysis of variance, ^∗∗^*P* < 0.01).

Tomato yellow leaf curl virus transmission, as indicated by the relative amount of TYLCV in tomato leaves exposed to one viruliferous adult *B. tabaci* Q female, was lower by females that contained both viruses (they were exposed to TSWV first) than by females that contained only TYLCV (*F*_1,14_ = 15.836, *P* = 0.00137) (**Figure 4**).

### Transmission of TYLCV by *B. tabaci* Q to TSWV-Infected Tomato Plants vs. Healthy Tomato Plants

**FIGURE 5:**
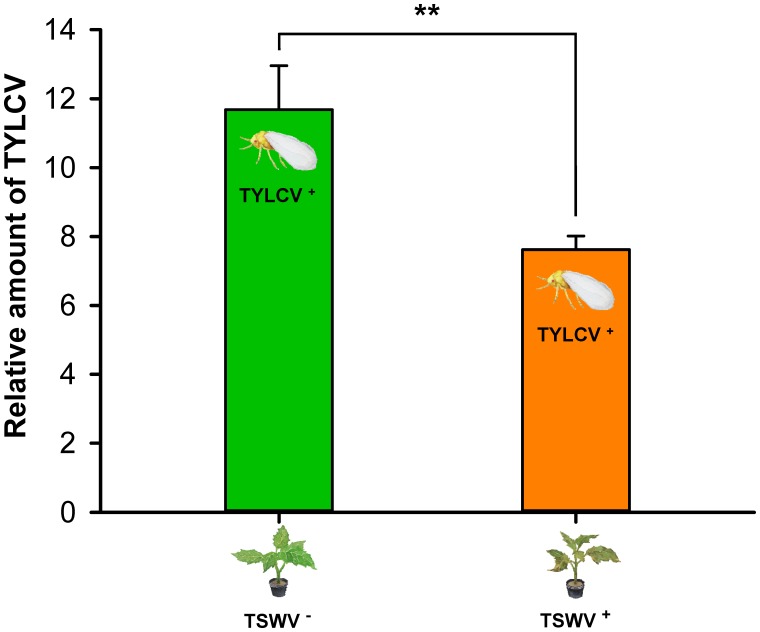
Transmission of TYLCV DNA to tomato plants by *Bemisia tabaci* Q females as affected by previous infection of the tomato plants by TSWV. TSWV^+^ or TSWV^-^: Tomato plants inoculated with TSWV or mock-inoculated, respectively, before they were exposed to *Bemisia tabaci* Q females that contained TYLCV. TYLCV^+^: Females that acquired TYLCV from another TYLCV-infected plant. Values are means ± SE. Asterisks indicate significant differences between TSWV^+^ vs. TSWV^-^ treatments (one-way analysis of variance, ^∗∗^*P* < 0.01).

As indicated by the relative quantities of TYLCV in the plants, the transmission of TYLCV by *B. tabaci* Q adult females (one female per plant) was lower to TSWV-infected tomato plants than to healthy tomato plants (*F*_1,14_ = 9.305, *P* = 0.00864) (**Figure 5**).

### Detection of TYLCV DNA in *F. occidentalis* Second-Instar Nymphs and Newly Emerged Adults

**FIGURE 6:**
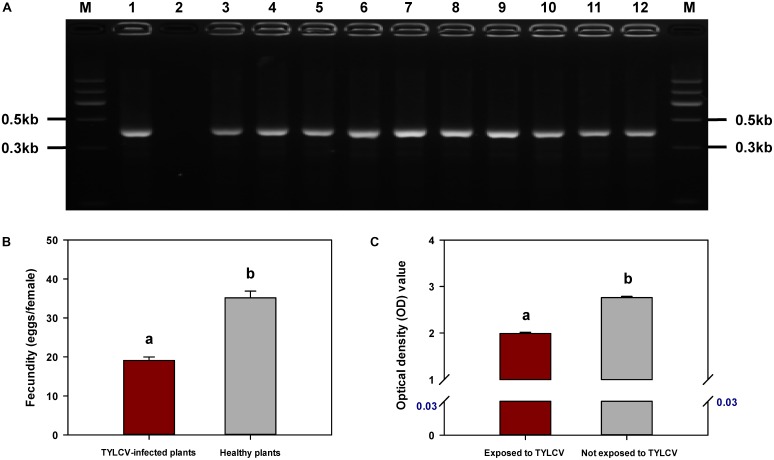
Acquisition of TYLCV by *Frankliniella occidentalis* **(A)**, fecundity of *F. occidentalis* as affected by TYLCV **(B)**, and transmission of TSWV by *F. occidentalis* as affected by previous exposure of the thrips to TYLCV **(C)**. **(A)** PCR amplification of TYLCV was performed with the DNA extracted from *F. occidentalis* second-instar nymphs (lanes 3–7; one nymph per lane) and newly emerged adults (lanes 8–12; one adult per lane) obtained from a TYLCV-infected tomato plant. Amplification products were visualized by agarose gel electrophoresis with Gelview staining. Lane 1: positive control; lane 2: negative control; lane M: Marker II (Tiangen). **(B)** Number of eggs laid by each female placed in a clip-cage attached to a leaf of a healthy or a TYLCV-infected tomato plant. Values are means ± SE of 10 replicate plants (three adults per plant). **(C)** The OD value of TSWV analysis obtained by DAS-ELISA indicated the difference between *F. occidentalis* that were exposed and not exposed to TYLCV. The number in blue indicates the mean OD value of healthy tomato plants. Values are means ± SE. For **(B,C)**, different letters indicate significant differences between treatments (*P* < 0.05).

Tomato yellow leaf curl virus was detected by PCR in all *F. occidentalis* second-instar nymphs that had fed on TYLCV-infected tomato plants and in all adults that emerged from those nymphs (**Figure 6A**).

### Fecundity of *F. occidentalis* Females on TYLCV-Infected and Healthy Tomato Plants

Fecundity was significantly lower for *F. occidentalis* females on TYLCV-infected tomato plants than on healthy tomato plants (*F*_1,18_ = 68.747, *P* < 0.001) (**Figure 6B**).

### Transmission of TSWV by *F. occidentalis* Females to Healthy Tomato Plants as Affected by Previous Exposure of Thrips to TYLCV-Infected Tomato Plants

The transmission of TSWV, as indicated by the amount of virus in tomato leaves exposed to one adult *F. occidentalis* female, was reduced if the thrips had been previously exposed to a TYLCV-infected tomato plant (*F*_1,22_ = 447.544, *P* < 0.001) (**Figure 6C**).

## Materials and Methods

### Plant Cultures, TSWV Inoculation, TYLCV Inoculation, and *B. tabaci* and *F. occidentalis* Populations

Pepper (*Capsicum annuum* L., cv Zhongjiao 6**)**, cotton (*Gossypium herbaceum* L., cv DP99B), and tomato (*Solanum lycopersicum* L., cv Zhongza 9) were grown as described previously (Chen et al., 2013). Pepper can be infected by TSWV and TYLCV, but cotton cannot be infected by TSWV and TYLCV. Hence, we chose pepper to be the virus host plant and chose cotton to be the control plant.

Tomato spotted wilt virus was maintained on *Datura stramonium*. The virus inoculum was prepared by grinding infected plant material in chilled 0.01 M phosphate buffer, pH 7, containing 1% (wt:vol) sodium sulfite and 2% (wt:vol) PVP. The inoculum was mechanically transmitted by rubbing the ground material onto the upper leaves of the pepper plants, which had been dusted with carborundum. Pepper plants were inoculated at the three-true-leaf stage. Pepper plants were assumed to be infected with TSWV when they developed characteristic symptoms that include plant stunting, bronzing, or chlorosis of leaves. Infection was then determined for inoculated and non-inoculated control plants by the molecular methods described in the next section. When these healthy and TSWV-infected plants had developed to the six–seven-true-leaf stage, they were used in the experiments. (Pan et al., 2013).

Tomato yellow leaf curl virus-infected tomato plants were obtained by carrier (*Agrobacterium tumefaciens*) -mediated inoculation using a TYLCV clone (GenBank accession ID: AM282874) that was initially isolated from tomato in China (Wu et al., 2006). TYLCV inoculum was prepared and plants were inoculated with TSWV as described by Chen et al. (2013). To account for the effects of the inoculation process, control treatments were mock-inoculated with a noncarrier buffer.

Since its collection in Beijing, China, in 2009, the *B. tabaci* Q population used in this research has been maintained on poinsettia plants (*Euphorbia pulcherrima* Wild. ex. Klotz.) in insect-proof screen cages under natural light and controlled temperature (26 ± 2°C) in a greenhouse. The purity of the *B. tabaci* Q population has been monitored as described by Chu et al. (2010).

Since its collection from melon (*Cucumis melo* L.) in a glasshouse at the Institute of Vegetables and Flowers, Chinese Academy of Agricultural Sciences (CAAS), Beijing, China, in 2003, the *F. occidentalis* population has been reared with fresh green bean pods Phaseolus vulgaris in a climate-controlled chamber (27 ± 1°C, 16 L: 8 days) (Zhang et al., 2013).

### RT-PCR Detection of TSWV in Pepper Plants and in *B. tabaci* Q

Total RNAs were extracted from pepper plants and *B. tabaci* Q using TRIzol reagent following the manufacturer’s protocol (Invitrogen). The resulting total RNA was resuspended in nuclease-free water and was quantified with a spectrophotometer (Thermo Scientific). Reverse transcription was then performed on 1.0 μg of each RNA sample, and the first-strand cDNA was synthesized using the PrimeScript RT reagent kit according to the manufacturer’s protocol (TaKaRa Biotechnology, Dalian Co., Ltd). Specific primers (forward primer NF302: 5′-5′GGGTCAGGCTTGTTGAGGAAAC-3′ and reverse primer NR575: 5′-TTCCCTAAGGCTTCCCTGGTG-3′) were used to detect TSWV (Mason et al., 2003). Polymerase chain reaction amplifications were performed in 20-μl reactions containing 1 μl of cDNA, 1 μl of each primer (10 μM each), 7 μl of ddH2O, and 10 μl of 2 × Tap10 Master Mix (Biosubstrate Technologies, Beijing, China). At the same time, PCR amplifications with negative control (template was ddH2O) and positive control (template was the tomato plant cDNA that contains TSWV) were performed. The cycling conditions were: 2 min of activation at 94°C followed by 30 cycles of 30 s at 94°C, 30 s at 60°C, and 30 s at 72°C; and a final extension of 5 min at 72°C. The PCR products were electrophoresed on a 1.5% agarose gel in a 0.5 × TBE buffer and were visualized by Gelview staining (Boonham et al., 2002; Pan et al., 2013).

### Acquisition of TSWV by *B. tabaci* Q

A pepper plant at the seven-true-leaf stage that had been inoculated with TSWV and that showed clear symptoms of TSWV infection was used as the virus source. The plant, which was enclosed in an insect-proof cage, was infested with approximately 200 newly emerged (0 to 24 h) *B. tabaci* Q females. Care was taken to transfer the whiteflies evenly onto all leaves of the TSWV-infected pepper plant. After AAPs of 3, 6, 12, 24, and 48 h, 10 adults were randomly collected from the top second and third leaves of the TSWV-infected pepper plant. During the collection of adults, care was taken not to disturb other whiteflies on the TSWV-infected pepper plant. Ten adults that had not been placed on the plant (0-h AAP) were also collected. The collected adults were stored at -80°C and were later individually assayed for TSWV RNA by RT-PCR as described in the previous section.

### Retention of TSWV RNA by *B. tabaci* Q

Approximately 200 *B. tabaci* Q adult females that had access to a TSWV-infected pepper plant for 72 h and that were known to contain TSWV (see **Table 1**) were placed on a healthy cotton plant, which is not a host plant of TSWV. One group of 10 live adults was collected at 5, 10, 15, and 20 days after placement. At day 14, the adults remaining on the cotton plant were transferred onto a new cotton plant so that the new adults that emerged from the progeny were not collected at day 15 and 20. The collected insects were stored at -80°C and were later individually assayed for TSWV RNA by RT-PCR.

### Quantification of TVLCV DNA by q-PCR

To quantify TYLCV acquisition and retention, the MiniBEST universal genomic DNA extraction kit ver.5.0, Cod: 9765 (TaKaRa Biotechnology, Dalian Co., Ltd.) was used to extract the DNA from female whiteflies. To quantify TYLCV in tomato plants, a plant genomic DNA kit (Tiangen Biotech, Beijing, Co., Ltd.) was used to extract the DNA from tomato leaves. **Table 2** lists the primers used for the quantification of TYLCV. The q-PCR reaction conditions for samples from plants were described by Pan et al. (2012), and those for samples from insects were described by Pakkianathan et al. (2015). TYLCV DNA was quantified with SuperReal PreMix Plus (SYBR Green) (Tiangen Biotech). Four technical replicates were amplified for each sample. The comparative cycle threshold 2^-ΔC_T_^ method was used to calculate the relative quantities of TYLCV.

**Table 2:** Primer sequences used for q-PCR analysis.

Gene or source of DNA	Primer	Primer sequence (5′ to 3′)	Reference
TYLCV	TY-F	GTCTACACGCTTACGCC	Pan et al., 2012
	TY-R	GCAATCTTCGTCACCC	
*Bemisia tabaci*	β-actin -F	TCTTCCAGCCATCCTTCTTG	Pakkianathan et al., 2015
	β-actin-R	CGGTGATTTCCTTCTGCATT	
Tomato	UBI-F	TCGTAAGGAGTGCCCTAATGCTGA	Pan et al., 2012
	UBI-R	CAATCGCCTCCAGCCTTGTTGTAA	


### Acquisition of TYLCV by *B. tabaci* Q Carrying TSWV

About 450 newly emerged *B. tabaci* Q adult females were placed in leaf clip-cages (50 adults per clip-cage) on the top second and third leaves (one clip-cage per leaf) of TSWV-infected pepper plants (at the six-true-leaf stage, about 2 weeks after virus inoculation) and healthy (mock-inoculated) pepper plants for a 72-h AAP. These adults were subsequently moved into replicate screen cages containing one TYLCV-infected tomato plant. There were 150 adults per replicate screen cage, with three replicate screen cages for adults from TSWV-infected pepper plants and three for adults from healthy pepper plants. After AAPs of 4, 12, 24, 36, 48, and 72 h, adults were randomly collected from symptomatic tomato leaves in each cage. Then, 20 females per replicate cage were stored at -80°C. The content of TVLCV DNA in these females was subsequently assessed by q-PCR as described later.

### Acquisition of TYLCV DNA by *B. tabaci* Q Females From Tomato Plants Inoculated in Different Order With Both TSWV and TYLCV

The acquisition of TYLCV by *B. tabaci* Q was tested as described earlier in this report, but the plants were inoculated with TYLCV and TSWV in different order. In the first case, we measured TYLCV acquisition from tomato plants that were first inoculated with TSWV and then inoculated with TYLCV 2 weeks later, or that were first mock-inoculated and then inoculated with TYLCV 2 weeks later. In the second case, we measured TYLCV acquisition from tomato plants that were simultaneously inoculated with TYLCV and TSWV, or that were simultaneously mock-inoculated and inoculated with TYLCV. In the third case, we measured TYLCV acquisition from tomato plants that were first inoculated with TYLCV and then inoculated with TSWV 2 weeks later, or that were first inoculated with TYLCV and were then mock-inoculated 2 weeks later. The content of TVLCV DNA in these females was subsequently assessed by q-PCR as described later.

### Retention of TYLCV DNA by *B. tabaci* Q Carrying TSWV

Newly emerged *B. tabaci* Q adult females were subjected to a 72-h AAP on TSWV-infected and healthy (mock-inoculated) pepper plants as described earlier. These adults were subsequently released into replicate screen cages containing one TYLCV-infected tomato plant as described earlier. After a 72-h AAP, the adult females in each replicate screen cage were transferred to a new replicate screen cage containing one healthy cotton plant, which is not a host of TYLCV. Twenty adult females were collected randomly from each screen cage after 0, 3, 6, 9, and 12 d and were stored at -80°C. The content of TVLCV DNA in these females was tested by q-PCR as described later.

### Transmission of TYLCV by *B. tabaci* Q Carrying TSWV

About 200 newly emerged *B. tabaci* Q adult females were placed in clip-cages (50 females per cage) on the top second and third leaves (one clip-cage per leaf) of TSWV-infected pepper plants (at the six-true-leaf stage, about 2 weeks after virus inoculation) and healthy (mock-inoculated) pepper plants. After a 72-h AAP, the adults were moved into insect-proof screen cages containing TYLCV-infected tomato plants (one plant and 100 adult females per cage). After a 72-h AAP, the adult females were collected and placed in clip-cages attached to healthy tomato plants with three true leaves; each plant had one clip-cage that contained one adult female. Each of two treatments (one female carrying TSWV or one control female) was represented by eight replicate clip-cages. After the clip-cages had been in greenhouse for 48 h, all of the whiteflies were removed, and the plants were kept in whitefly-proof screen cages. After 10 d, the first two youngest leaves of each tomato plant were collected (Mason et al., 2008; Péréfarres et al., 2011), and the DNA in the leaves was extracted and tested for TYLCV by q-PCR as described later.

### Transmission of TYLCV by *B. tabaci* Q to TSWV-Infected Tomato Plants vs. Healthy Tomato Plants

About 200 newly emerged *B. tabaci* Q adult females were moved into insect-proof cages containing TYLCV-infected tomato plants (one plant and 100 adult females per cage). After a 72-h AAP, the adult females were collected and placed in clip-cages attached to TSWV-infected tomato plants and healthy tomato plants with three true leaves; each plant had one clip-cage that contained one adult female. Each of the two treatments (TYLCV-viruliferous adults on TSWV-infected tomato plants or on healthy tomato plants) was represented by eight replicate clip-cages. After the clip-cages had been in place for 48 h, all of the whiteflies were removed, and the tomato plants were kept in whitefly-proof screen cages. After 10 d, the first two youngest leaves of each tomato plant were collected (Mason et al., 2008; Péréfarres et al., 2011), and the DNA was extracted from the tomato leaves and tested for TYLCV by q-PCR as described later.

### Acquisition of TYLCV by *F. occidentalis*

A tomato plant at the seven-true-leaf stage that had been inoculated with TYLCV and that showed clear symptoms of TYLCV infection was used as the virus source. The plant, which was enclosed in a thrips-proof screen cage, was infested with approximately 200 *F. occidentalis* adults. Adult females were allowed to oviposit on the tomato plant for 2 days. The second-instar nymphs and the newly emerged adults (0 to 24 h) were then collected and later assayed for TYLCV DNA by PCR as described by Ghanim et al. (2007). This was performed with five replicate TYLCV-infected plants.

### *F. occidentalis* Fecundity as Affected by TYLCV

One newly emerged adult female *F. occidentalis* was collected from a colony feeding on healthy bean pods. The adult was placed in a clip-cage attached to a leaf of a healthy (mock-inoculated) or TYLCV-infected tomato plant; each plant had three clip-cages, and each treatment was represented by 10 replicate plants. Seven days later, these infested leaves were detached, and the number of live nymphs and eggs were counted using a stereomicroscope. The data from the three infested leaves from each plant were pooled and treated as one biological replicate (*n* = 10). *F. occidentalis* fecundity was reported as the mean number of offspring per thrips.

### Transmission of TSWV by *F. occidentalis* Carrying TYLCV

Newly hatched *F. occidentalis* nymphs (0–6 h since hatching) were placed in a thrips-proof cage (50 nymphs per cage) containing a TYLCV-infected tomato plant or a healthy (mock-inoculated) tomato plant for a 24-h AAP. These nymphs were then transferred to another cage containing a TSWV-infected tomato plant for a 48-h AAP. The transmission of TSWV by the adults was then assessed by a leaf disk assay (with leaf disks from a healthy tomato plant) as previously described (Wijkamp and Peters, 1993; Tedeschi et al., 2001). Each of the two treatments was represented by 12 replicates. In addition, the concentration of TSWV in leaf disk extracts was assessed by using a DAS-ELISA kit supplied by Adgen Phytodiagnostics (Neogen Europe (Ayr), Ltd.) and by following the manufacturer’s recommendations. Absorbance and optical density (OD) were determined with a fluorescence microplate reader (SpectraMax M2e, Molecular Devices) at a wavelength of 405 nm.

### Data Analysis

One-way analyses of variance (ANOVAs) were used to determine whether treatment effects were significant. SPSS 17.0 (SPSS Inc., Chicago, IL, United States) was used for all statistical analyses.

## Discussion

Although *B. tabaci* Q cannot transmit TSWV (Zhao et al., 2015), we showed in this study that it can acquire and retain TSWV. In the interaction between TSWV, *B. tabaci* Q, and host plants, it follows that any effects of TSWV on *B. tabaci* Q are direct, i.e., do not result from the changes in the host plant that could occur following TSWV transmission. Previous exposure of *B. tabaci* Q to TSWV reduced the whitefly’s ability to acquire, retain, and transmit TYLCV (**Figures 1, 3, 4**), and the whitefly’s ability to acquire TYLCV from tomato plants was reduced if the plants were inoculated with TSWV before TYLCV or concurrently with TYLCV (**Figures 2A,B**). Previous exposure of tomato plants to TSWV reduced the transmission of TYLCV to those plants by *B. tabaci* Q (**Figure 5**). We also found that *F. occidentalis* can acquire TYLCV (**Figure 6A**) and that its fecundity was lower on TYLCV-infected tomato plants than on healthy tomato plants (**Figure 6B**). Moreover, the transmission of TSWV by *F. occidentalis* was reduced when the thrips had previously fed on TYLCV-infected plants (**Figure 6C**). These results are consistent with our hypothesis that a persistently transmitted virus can suppress the transmission of a second virus by decreasing some fitness characteristics of its vector and by competing with the second virus in the host plant.

The current study provides additional evidence that transmission efficiency can be significantly affected by changes in vector fitness. Previous research has indicated that virus transmission can be affected by insect vector characteristics; TYLCV-carried whiteflies fed more readily than noncarried whiteflies, and spent more time salivating into sieve tube elements (Liu et al., 2013). Similarly, the potato leafroll virus infection of potato alters the feeding behavior of its aphid vector (Alvarez et al., 2007). The transmission of TYLCV, for example, differs among *B. tabaci* biotypes (Chu et al., 2006; Pan et al., 2012; Ning et al., 2015). Virus transmission is also often greater for female than for male piercing-sucking insect vectors (Ghanim and Czosnek, 2000). For example, female adults of the planthopper *Peregrinus maidis* are more efficient vectors of the rice stripe virus than male adults (Narayana and Muniyappa, 1996), and female adults of *B. tabaci* Q are more efficient vectors of TYLCV than male adults (Ning et al., 2015). In some cases, however, male insects are more efficient vectors. Male thrips, for example, are more efficient vectors than female thrips (van de Wetering et al., 1999; Stafford et al., 2011). Transmission can also be affected by endosymbiotic bacteria that produce a GroEL homolog, which seems to protect begomoviruses in the *B. tabaci* hemolymph (Morin et al., 1999, 2000; Gottlieb et al., 2010; Himler et al., 2011). The transmission of TYLCV by *B. tabaci* is enhanced by the endosymbiotic bacterium *Hamiltonella* sp. (Gottlieb et al., 2010; Su et al., 2013). Transmission is also affected by immune responses such as autophagy; the activation of autophagy significantly reduces TYLCV (Medeiros et al., 2004; Wang et al., 2016).

Persistently transmitted viruses can substantially increase the fitness of their vectors and thereby increase virus transmission (Mauck et al., 2012). For example, TYLCV can improve the performance of *B. tabaci* Q, including their body size, longevity, fecundity, and so on (Pan et al., 2012; Chen et al., 2013); TSWV infection increases *F. occidentalis* oviposition and makes them develop faster (Maris et al., 2004); and persistently transmitted viruses enhance aphid performance (including in terms of growth, reproduction, and survival) on infected plants (Montllor and Gildow, 1986; Castle and Berger, 1993; Jiménez-Martínez et al., 2004a). Our previous results indicated that TSWV can reduce the longevity and fecundity of *B. tabaci* on pepper plants (Pan et al., 2013). The current results revealed that prior feeding on TSWV-infected plants reduces the ability of *B. tabaci* Q to acquire, retain, and transmit TYLCV and that prior feeding on TYLCV-infected plants reduces *F. occidentalis* fecundity and its ability to transmit TSWV. In other words, the current study provides additional evidence that transmission efficiency can be significantly affected by changes in vector fitness.

The results of the current study have several possible explanations. We have two hypotheses. First, the ingestion of a persistently transmitted virus (virus 1) may activate autophagy in the vector (vector 2) of a second virus (virus 2), which would decrease the titer and gene expression of virus 2 in vector 2. This is possible because autophagy, which is an intrinsic antiviral process, can decrease the infection of a persistently transmitted plant virus by reducing the amount of viral coat protein and DNA (Wang et al., 2016). This hypothesis is related to the insect’s immune response “memory” or prolonged immune response to a virus that can interfere in another virus. Second, the presence of virus 1 in vector 2 could induce changes in the feeding behavior of vector 2, thereby affecting the acquisition of virus 2. Determining whether either of these explanations is correct will require additional research.

The objective of this study was to assess the effects of one persistently transmitted virus on the transmission of another persistently transmitted virus. The results are consistent with the view that, while persistently transmitted viruses can substantially increase the fitness (including longevity, body size, fecundity) of their vectors, they can also decrease the fitness of vectors of other viruses. These characteristics may help the vector population of persistently transmitted viruses to spread in the field, where there is significant competition among persistently transmitted viruses and other pathogens for host resources.

## Author Contributions

GC contributed to the chemical ecological laboratory work, performed the major part of entomological manipulations, participated in data analysis, conception and design of the study, and drafting of the manuscript. QS carried out the ecological manipulations. XS contributed to viral and microbiological manipulations. HP contributed to insect and plant husbandry and data collection. XJ carried out the statistical analyses. YZ contributed to the conception and design of the study, coordinated the study, and helped in drafting the manuscript. All authors gave final approval for publication.

## Conflict of Interest Statement

The authors declare that the research was conducted in the absence of any commercial or financial relationships that could be construed as a potential conflict of interest.
